# Reversal of portal gastropathy with partial internal biliary diversion in a case of progressive familial intrahepatic cholestasis

**DOI:** 10.5339/qmj.2021.45

**Published:** 2021-10-08

**Authors:** Arkadeep Dhali, B Rathna Roger, Elaina Pasangha, Christopher D'Souza, Gopal Krishna Dhali

**Affiliations:** ^1^Department of GI Surgery, IPGME&R, School of Digestive & Liver Diseases, Kolkata, India; ^2^Department of Critical Care Medicine, St John's National Academy of Health Sciences, Bangalore, India; ^3^Department of Critical Care Medicine, Narayana Hrudayalaya, Bangalore, India E-mail: arkadipdhali@gmail.com; ^4^Department of Gastroenterology, IPGME&R, School of Digestive & Liver Diseases, Kolkata, India

**Keywords:** progressive familial intrahepatic cholestasis, portal gastropathy, end stage liver disease, partial internal biliary diversion, molecular chaperones

## Abstract

Progressive intrahepatic cholestasis is a rare, genetic disorder causing bile acid secretion or transport defects. It can result in intrahepatic cholestasis that can progress to end-stage liver disease. Diagnosis is made using a combination of clinical and biochemical approaches. Genetic testing is currently the gold standard for investigation.

We report a case of an 18-month-old male child with cholestatic pattern of jaundice from 16 months of life, which was associated with features suggestive of portal gastropathy. Detailed workup led to the diagnosis of progressive intrahepatic cholestasis (type 2). Early diagnosis prevented the need for liver transplant, and the child underwent surgical treatment with partial internal biliary diversion. Portal gastropathy and disease progression dramatically changed with corrective surgery. The patient was symptom-free at 10-week follow-up.

Detecting this rare genetic disorder early has very good therapeutic implications from the patient's perspective and their morbidity and mortality profile; if untreated, it has a high propensity to progress to end-stage liver disease. The requirement of surgical interventions and liver transplantation is individualized on a case-to-case basis. An early diagnosis and initiation of treatment can prevent the need for a liver transplant as shown in the present case.

## Introduction

Diagnosis and treatment of pediatric cholestatic liver disease is a challenge. A multidisciplinary approach and genetic testing are required for a definitive diagnosis. Progressive familial intrahepatic cholestasis is a hereditary, autosomal recessive liver disorder causing bile acid secretion or transport defects, which often progresses to end-stage liver disease (ESLD). It accounts for 10%–15% cases of neonatal cholestasis syndrome and 10%–15% of children requiring liver transplantation.^1,2^ 1It occurs in 1 per 50,000 to 1 per 100,000 births although the exact prevalence is not known. The disease affects both genders equally and has been reported worldwide.^3,4^ Although this condition is known to cause significant morbidity and mortality, it has not been adequately investigated in India.^5,6^ Understanding the disease spectrum in Indian children is limited by the lack of diagnostic facilities (genetic analysis and immunostaining).^7^ Moreover, establishing an accurate and timely diagnosis has direct therapeutic implications for this subset of patients, as it progresses to ESLD; hence, the onus is on the treating physician to come to a correct conclusion.

## Case report

We present a case of an 18-month-old boy, born of a non-consanguineous marriage and by normal vaginal delivery, with a birth weight of 3 kg and an uneventful perinatal period. The child had normal development and was fully immunized for age till then. The mother had no history of fever, jaundice, and pruritis during the antenatal period. The child presented with yellowish discoloration of the eyes and urine for 3 months and disabling pruritis for 2 months. The mother also recalled four episodes of bilateral nose bleeds in the past 3 months and occasional gum bleeds. She had also noticed that child was passing black tarry stool for the last 2 weeks.

On physical examination, no dysmorphic features were found, and the child weighed 8.5 kg (-2.11 Z-score), stand 74 cm ( − 2.46 Z-score), and appeared pale and icteric. His abdomen was distended with tenderness in the right upper quadrant. Veins and sinuses were not dilated. Multiple pruritic scar marks were seen. The edge of the liver edge was palpable 4 cm below the right costal margin. Ophthalmology opinion was taken for examination of Kayser Fleischer rings, and the result was negative.

Initial laboratory findings (Table 1) revealed severe microcytic hypochromic anemia with hemoglobin of 5.6 g/dL and elevated liver enzymes [alkaline phosphatase (ALP), 1589 U/L; aspartate transaminase (AST), 288 U/L; alanine transaminase (ALT), 173 U/L; gamma-glutamyl transferase (GGT), 84 U/L; albumin, 47 g/L; lactate dehydrogenase (LDH), 132 IU/L; total bilirubin, 14.8 mg/dL; conjugated bilirubin, 12.2 mg/dL; serum bile acid, 170 mol/L; vitamin D3, 6.91 ng/mL]. Serum electrolytes, calcium, magnesium, phosphate, urea and creatinine, thyroid stimulating hormone, and C-reactive protein were within the normal limits. The serum lipase level was 34 U/L. Hemoglobinopathy screening was performed to investigate the cause of jaundice, and the result was negative. Iron panel confirmed iron deficiency (serum iron, 4 mol/L; (total iron-binding capacity, 90 mol/L; transferrin saturation, 0.04%; and ferritin, 5 g/L). Serum protein electrophoresis was performed to rule out autoimmune hepatitis, which did not show dominant protein bands. Alpha-1 antitrypsin level was 2.2 μmol/L. Results of the serological workup for infective etiologies (such as hepatitis B, hepatitis C, Epstein-Barr virus, cytomegalovirus, parvovirus B19, and syphilis) were negative. Three urine samples were sent for non-reducing sugars, and 2 of 3 were positive. To rule out metabolic causes of infantile jaundice, metabolic screening was performed for tyrosinemia, galactosemia, and hereditary fructose intolerance, and the result was negative.

**Figure 1.**
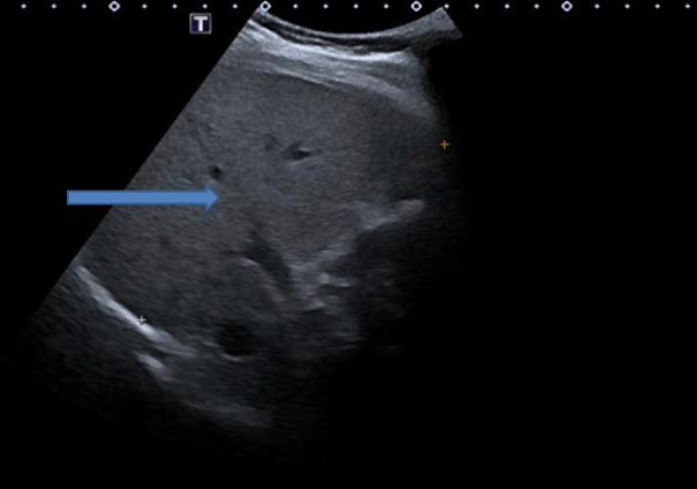
Ultrasonography of the abdomen revealed enlarged size of the liver with increased nodularity (blue arrow)

Ultrasonography (USG) of the abdomen revealed an enlarged liver with increased nodularity (Figure 1). Gross ascites and mild splenomegaly were present. USG showed normal gallbladder, common bile duct, and portal veins. Findings of magnetic resonance cholangiopancreatography were normal. For severe anemia, he was transfused with two units of packed cells, and the post-transfusion hemoglobin level was 9.3 g/dL.

**Figure 2.**
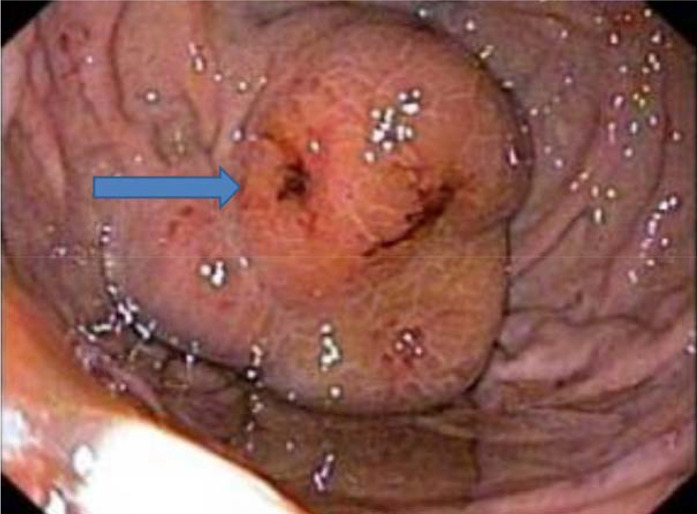
Endoscopic image of gastric fundal varices (blue arrow)

**Figure 3.**
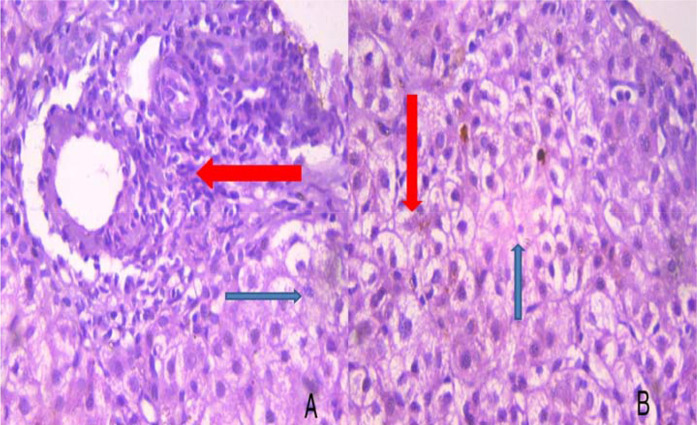
Hematoxylin and eosin-stained liver biopsy (A: 100 × , B: 400 × ): intrahepatic cholestasis with moderate fibrosis (blue arrow) and inflammatory cells (red arrow)

To evaluate the cause of melena, upper gastrointestinal endoscopy was performed under general anesthesia, which revealed gastric fundal varices (Figure 2), and four bands were applied. He was started on ursodeoxycholic acid, ondansetron, and rifampicin for pruritus. Vitamin A, D, E, and K supplementations were given. Spironolactone and nadolol were added for portal hypertension. Paracentesis was performed on the third day of admission. Peritoneal fluid cultures were negative for tuberculosis, bacterial growth, or fungus. Biochemical analysis of peritoneal fluid was not contributory and was negative for malignant cells. As the etiology of jaundice was still unclear, USG-guided liver biopsy was taken, which showed intrahepatic cholestasis with moderate fibrosis and inflammatory cells (Figure 3); hence, a preliminary diagnosis of progressive intrahepatic cholestasis (PFIC) was made.

Considering the possibility of an inherited disorder, genetic tests for ABCB11, ABCB4, ATP8B1, JAG1, NOTCH2, and TJP2 were performed and revealed positive results for ABCB11 confirming the diagnosis to be PFIC type 2.

After getting the definitive diagnosis, the parents were counseled regarding the surgical treatment options, which included nasobiliary drainage, partial external biliary diversion (PEBD), partial internal biliary diversion (PIBD), ileal exclusion, and liver transplantation. After obtained informed consent, the child was taken for PIBD with a cholecystojejunocolic anastomosis. Intraoperatively, a 10-cm loop of bowel was isolated from the mid jejunum, and this conduit was sutured in an isoperistaltic manner, inferiorly to the anterior aspect of the ascending colon and superiorly to the gall bladder. The full thickness of gallbladder was anastomosed to the serosa of the conduit in a single layer. A single layer of serosa-to-serosa anastomosis was performed between the conduit and the colon. The distal end of the jejunum was tapered, and an intussuscepted nipple valve was introduced prior to anastomosing to the colon to prevent colonic contents from entering the conduit. The postoperative period was uneventful, and the child was discharged on postoperative day 12 on oral medications.

Jaundice and pruritus were assessed by 5-D itch score in the postoperative period and follow-up visits, which showed marked reduction in pruritis. On 10 weeks follow-up, the patient was asymptomatic, with biochemical investigations (Table 2) showing total bilirubin level of 3 mg/dL, conjugated bilirubin of 1.7 mg/dL, ALP of 250 U/L, AST of 42 IU/L, ALT of 65 IU/L, and serum bile acid of 60 μmol/l. In addition, the child was gaining weight and height. Although parents complained of intermittent diarrhea, it can be attributed to the high bile salt content in the intestine.

## Discussion

Elimination of bilirubin in bile is affected in multiple genetic disorders that work through several mechanisms. The abnormality of biliary excretion is shared with excretory defects of all or some organic anions. These commonly result from mutations (Table 3) in the following genes: *SERPINAI* (alpha1-antitrypsin deficiency), *JAG1* (alagille syndrome), *ATP8B1* (PFIC-1), *ABCB11* (PFIC-2), *ABCB4* (PFIC-3), and *MRP2* (Dubin–Johnson syndrome). Conditions such as PFIC and benign recurrent intrahepatic cholestasis, albeit very rare, cause conjugated hyperbilirubinemia because of reduced bile flow. Type 1 PFIC is characterized by a defect in aminophospholipid translocase protein that maintains canalicular membrane stability. Types 2 and 3 have defect in bile acid transporter and phospholipid translocase, respectively. This presentation in early childhood limits the number of possible differentials to the abovementioned conditions. Since a genetic testing (deficiency of BSEP) was performed, the diagnosis was PFIC 2. The most commonly seen form of PFIC is severe BSEP deficiency. A severe deficiency of this protein usually presents very early, in the first few months of life, and patients have elevated levels of liver enzymes and bile acids and have a deficiency of fat-soluble vitamins. The serum levels of GGT are normal.^8,9^ Although pruritis and jaundice are common presentations for the disease, portal gastropathy as a presenting complaint is not reported previously. The severity of pruritis, age at presentation, and genetic findings point toward the diagnosis of PFIC type 2. A PubMed-based literature search using the MeSH words “progressive familial intrahepatic cholestasis” and “portal gastropathy” was performed to analyze the work completed in this field. The results indicated that it has a high propensity to progress to ESLD, but the rapidity of the progression to ESLD supported by portal gastropathy clinically and imaging was not documented previously. Our patient was treated in line with the management protocol previously described. The child was started on rifampicin, ursodeoxycholic acid for pruritis, spironolactone, and nadolol for portal hypertension, and endoscopic band ligation was performed for the bleeding varices. Although these medications provided relief of pruritic symptoms, they have no role in stopping the disease progression.^3,4^ Therefore, surgical interventions such as ileal bypass, PEBD, and PIBD are performed to reduce the circulating bile acids.^10,11,12^ Agarwal et al. described 24 PFIC cases where pruritis in two-thirds of the cases had resolved with medical management, three requiring biliary diversion with dramatic improvement and one improved after liver transplant.^7^ In another case series, Flores et al. demonstrated two patients with PFIC who underwent an internal diversion procedure (conduit between the gallbladder and transverse colon) had resolution of pruritis without progression of liver disease.^13^ As the disease had progressed and resulted in portal gastropathy leading to gastric varices, the progression to ESLD had already occurred. Although a band ligation was performed in this case to prevent rebleeding, as Ng et al. described, a significant number of these patients will have rebleeding and will need a therapeutic liver transplant possibly at a later stage in their life to cope with the disease.^14^ Therefore, selecting a treatment modality in the case of PFIC is much debatable, i.e., whether to go for a diversion procedure or for liver transplant; however, ultimately, liver failure and persistent pruritis may point to the need for liver transplantation.^9^ More studies and long-term follow-up are necessary before universal recommendation.

Furthermore, a new modality emerged in the treatment of these genetic conditions. Molecular chaperones maintain the tertiary structure of mutated protein molecules and thereby enhance their intracellular trafficking to cell membranes. These are potential therapeutic options for those with PFIC with these specific mutations. This approach is similar to membrane trafficking of cystic fibrosis transmembrane conductance regulator (CFTR) 2in cystic fibrosis,^15^ and 4-phenylbutyrate, one such chaperone, was proposed to reverse the symptoms and biochemistry of cholestasis in PFIC. This was demonstrated in several patients with PFIC types 1 and 2 who had missense mutations.^16,17^

## Conclusion

Pediatric cholestatic liver disease is challenging to both diagnose and treat. Its presentation should raise strong clinical suspicion to look for the presence of genetic diseases, and a complete evaluation is necessary. Detecting progressive familial intrahepatic cholestasis early has very good therapeutic implications from the patient's perspective and their morbidity and mortality profile, as it has a high propensity to progress to ESLD if untreated. The requirement of surgical interventions and liver transplantation is individualized on a case-to-case basis. This case highlighted that an early diagnosis and initiation of treatment such as biliary diversion procedures not only provides relief from pruritus but also reverse portal hypertension. This can prevent the need for a liver transplant.

### Conflicts of interest

None declared.

### Financial support

Nil.

### Declaration of patient consent

Written informed consent was obtained from the patient for publication of this case report and accompanying images. A copy of the written consent is available for review by the Editor-in-Chief of this journal on request.

### Ethical committee Approval

Institutional ethics committee granted permission to publish the anonymous case report.

## Figures and Tables

**Table 1:** Baseline laboratory investigations

Laboratory investigations	Results	Normal range

Hemoglobin	5.6 g/dL	13.8 to 17.2 g/dL

Alkaline phosphatase	1589 U/L	

Aspartate transaminase	288 U/L	8 to 33 U/L

Alanine transaminase	173 U/L	4 to 36 U/L

Gamma-glutamyl transferase(GGT)	84 U/L	9 to 48 U/L

Albumin	47 g/L	34 to 54 g/L

Lactate dehydrogenase	132 IU/L	140 to 280 U/L

Total bilirubin	14.8 mg/dL	0.3 to 1.2 mg/dL

Conjugated bilirubin	12.2 mg/dL	0.1 to 1.2 mg/dL

Serum bile acids	170 μmol/L	11 μmol/L

Vitamin D3	6.91 ng/mL	20 to 40 ng/mL

Calcium	9.8 mg/dL	8.6 to 10.3 mg/dL

Magnesium	1.9 mg/dL	1.7 to 2.2 mg/dL

Phosphate	1.6 mg/dL	3.4 to 4.5 mg/dl

Urea	12.3 mg/dL	5 to 20 mg/dL

Creatinine	0.83 mg/dL	0.74 to 1.35 mg/dL

Thyroid-stimulating hormone	1.2 mIU/L	0.5 to 5.0 mIU/L

C-reactive protein	1.4 mg/L	< 3.0 mg/L

Serum lipase	34 U/L	10 to 140 U/L

Serum iron	4 μmol/L	10.74 to 30.43 μmol/L

TIBC	90 μmol/L	42.96 to 80.55 μmol/L

Transferrin saturation	0.04%	15% to 50%

Ferritin	5 μg/L	11 to 307 μg/L

Alpha-1 antitrypsin	2.2 μmol/L	20 to 53 μmol/L


**Table 2:** Comparison of pre- and postoperative biochemical parameters

LAB INVESTIGATION	PRE-OP VALUES	POST-OP VALUES

Total bilirubin	14.8 mg/dL	3 mg/dL

Conjugated bilirubin	12.2 mg/dL	1.7 mg/dL

ALP	1589 U/L	250 U/L

AST	288 IU/L	42 IU/L

ALT	173 IU/L	65 IU/L

Serum bile acids	170 μmol/L	60 μmol/L


**Table 3:** Genetic profiling of diseases causing neonatal cholestasis

GENE	DISEASE

SERPINAI	Alpha1-antitrypsin deficiency

JAG1	Alagille syndrome

ATP8BI (PFIC)-1	Progressive intrahepatic cholestasis

ABCB11 (PFIC-2)	Progressive intrahepatic cholestasis

ABCB4 (PFIC-3)	Progressive intrahepatic cholestasis

MRP2	Dubin-Johnson syndrome

